# Highly Accurate Pneumatically Tunable Optofluidic Distributed Feedback Dye Lasers

**DOI:** 10.3390/mi15010068

**Published:** 2023-12-29

**Authors:** Hongtao Feng, Jiaxin Zhang, Weiliang Shu, Xiaosong Bai, Liang Song, Yan Chen

**Affiliations:** Shenzhen Institute of Advanced Technology, Chinese Academy of Sciences, Shenzhen 518055, China; ht.feng@siat.ac.cn (H.F.); jx.zhang1@siat.ac.cn (J.Z.); wl.shu@siat.ac.cn (W.S.); xs.bai@siat.ac.cn (X.B.); liang.song@siat.ac.cn (L.S.)

**Keywords:** tunable DFB laser, optofluidic chip, photonic device

## Abstract

Optofluidic dye lasers integrated into microfluidic chips are promising miniature coherent light sources for biosensing. However, achieving the accurate and efficient tuning of lasers remains challenging. This study introduces a novel pneumatically tunable optofluidic distributed feedback (DFB) dye laser in a multilayer microfluidic chip. The dye laser device integrates microfluidic channels, grating structures, and vacuum chambers. A second-order DFB grating configuration is utilized to ensure single-mode lasing. The application of vacuum pressure to the chambers stretches the soft grating layer, enabling the sensitive tuning of the lasing wavelength at a high resolution of 0.25 nm within a 7.84 nm range. The precise control of pressure and laser tuning is achieved through an electronic regulator. Additionally, the integrated microfluidic channels and optimized waveguide structure facilitate efficient dye excitation, resulting in a low pump threshold of 164 nJ/pulse. This pneumatically tunable optofluidic DFB laser, with its high-resolution wavelength tuning range, offers new possibilities for the development of integrated portable devices for biosensing and spectroscopy.

## 1. Introduction

Optofluidic devices have become important platforms for miniaturized and multifunctional optical systems [1,2,3,4]. These devices seamlessly integrate a diverse range of optical components, such as waveguides, light sources, and lenses [5,6,7,8]. An integrated light source holds significant promise in lab-on-a-chip applications, including on-chip plasmonic sensing, optical and plasmonic trapping, and optofluidic manipulation [9,10,11]. Notably, optofluidic dye lasers have emerged as a promising technology for the advancement of innovative photonic devices and highly sensitive biochemical sensors [12,13]. By integrating microcavities, microfluidic channels, and liquid gain media, these lasers can generate miniature coherent light sources directly on a microchip [14,15,16,17,18,19]. Laser-based systems prove invaluable for characterizing biomolecular interactions through the measurement of wavelength shifts in photonic resonators [20,21,22]. In particular, the tunability of optofluidic lasers would greatly enhance various areas of biomedical research, with a special focus on biosensing and diagnostic applications [23].

A dynamically tunable light source is a highly significant and critically needed technology [24,25,26,27], as it not only eliminates the time-consuming process of repeatedly replacing light sources, but also enables precise real-time control over the adjustment of the laser output wavelength. This satisfies the demand for diverse and flexible light sources in lab-on-a-chip applications. The flexible configuration of microfluidic systems enables diverse designs to modulate optofluidic dye lasers [28]. Several tuning methods have been demonstrated, such as varying the refractive indices of dye solutions or modifying the microcavities [29,30]. However, the need for the frequent replacement of dye solutions and grating periods severely hampers real-time dynamic tuning in optofluidic dye lasers. Li et al. reported a method of continuously stretching and compressing a microfluidic chip on a mechanical stage [31,32]. Nevertheless, the deformation of the entire device using a mechanical stage impedes integration with more complex microfluidic systems. Song et al. presented a pneumatic tuning approach using an air-gap etalon for achieving tunability in optofluidic dye lasers, enabling the straightforward implementation of optical cavity tuning [33]. Among the various reported dye laser resonator designs, distributed feedback (DFB) gratings remain highly favorable for achieving narrow linewidth and single-mode lasing with facile fabrication. This makes DFB lasers well suited for integration into microfluidic chips for a variety of applications.

The development of stretchable microfluidic chips has gained significant attention due to their advantages in providing innovative solutions for flexible and adaptable biomedical devices [34,35]. For instance, hydrodynamic manipulation in microfluidics can effectively separate particles by size, and when coupled with external mechanical tuning, can enhance separation performance [36,37,38]. However, the application of external mechanical forces often poses challenges to the stability and reliability of the chips, which can interfere with other integrated functional units. As a result, researchers are now exploring the development of microfluidic chips that enable precise tunability and localized deformation. The emergence of organ-on-a-chip technology has significantly advanced the recreation of human physiological systems on microfluidic platforms [39]. To mimic physiological motions like breathing, peristalsis, and vascular biomechanics, a precisely controlled mechanical stretching method was developed to meet the demands of organ chips [39,40,41]. This method inspired us to develop a novel strategy to integrate vacuum chambers with thin-film DFB structures as a dye laser tuning mechanism. This integration offers improved precision and efficiency in dye laser generation and wavelength tuning.

In this study, we present an optofluidic dye laser based on a DFB configuration. This laser design offers highly sensitive and accurate modulation capabilities. The Bragg condition is given by the following: $\lambda =2n_{eff}\Lambda /m$, where λ is the lasing wavelength, $n_{eff}$ is the effective refractive index of the waveguide mode, Λ is the grating period, and *m* is the Bragg order. Second-order DFB lasers (Bragg order *m* = 2) are of considerable research interest due to their capacity for realizing laser output in both horizontal and vertical directions, thus exhibiting significant potential. The microfluidic chip we developed integrates a second-order DFB dye laser, providing a compact light source suitable for a wide range of applications. Compared to a first-order DFB laser, the second-order configuration offers a key advantage in that it can produce single-mode output without requiring an additional phase shift section in the grating. This simplified design and fabrication process make second-order DFB lasers highly compatible with optofluidic platforms and lab-on-a-chip devices. Additionally, to achieve precise tunability, we implement a pneumatic stretching technique to modulate the grating period on a thin film. This approach utilizes an electronic vacuum regulator to control the force applied to the thin film with great precision. This method ensures that a high-performance single-mode laser output is maintained throughout the tuning process, making it a reliable light source for microfluidic systems. By incorporating mirror treatments, the pump laser can efficiently excite dye molecules within the microfluidic channel, while the output laser is readily collected by a spectrometer for analysis. Our integrated approach for constructing a tunable laser light source within a microfluidic chip offers great potential for miniaturized setups and cost-effective mass production, opening up new horizons for the application of various novel optical systems.

## 2. Materials and Methods

### 2.1. Device Design

**Figure 1:**
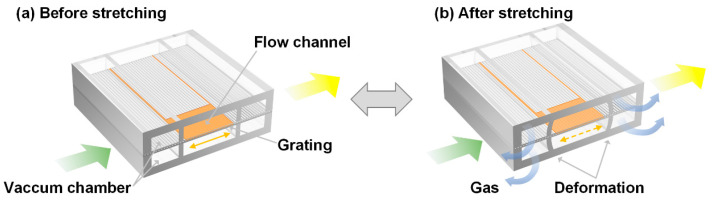
Schematic design of the optofluidic dye laser device and its tuning mechanism. The laser wavelength is adjusted before (**a**) and after (**b**) stretching the grating membrane using vacuum force. Green: Pump light, Yellow: Laser output. Orange line with dual arrows highlights the deformation of the grating film.

A second-order DFB dye laser was embedded in a microfluidic chip using a novel fabrication strategy. The optofluidic dye laser device consists of a grating layer, a flow channel layer, and the vacuum chambers on both sides (the bottom and top chambers are interconnected as a unified vacuum chamber via a membrane hole, not presented in Figure 1). The channel contained one-dimensional grating structures with a 417 nm period and 100 nm depth, and the active dye solution with Rhodamine 6G was filled in the channel. The refractive index (RI) of the active solution (RI = 1.42) is slightly higher than the surrounding PDMS channel (RI = 1.412), forming the laser resonator. High-quality lasing performance is achieved when pumped with a 532 nm laser. The laser wavelength is tuned by pneumatically stretching the grating period on a thin film, with the precise control of the applied force via an electronic vacuum regulator, as illustrated in Figure 1.

### 2.2. Fabrication

**Figure 2:**
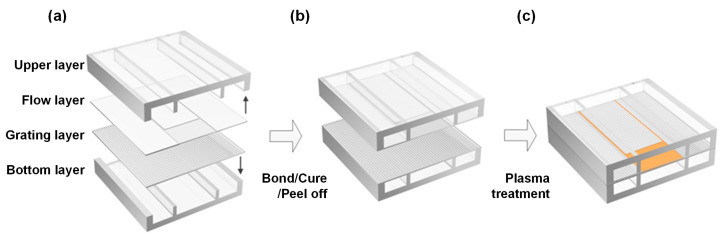
The schematic fabrication process of optofluidic dye laser devices. The optofluidic device consists of four layers (**a**) that are bonded sequentially (**b**), and it contains four lateral vacuum chambers (**c**).

The optofluidic dye laser device was fabricated from flexible polydimethylsiloxane (PDMS, RTV 615, GE, New York, NY, USA) using a series of soft lithography techniques. Figure 2 illustrates a brief schematic fabrication procedure. The upper layer consisted of 150 µm high microchambers and was bonded to a 1.2 µm high flow layer. The bottom layer had the same structures as the upper layer and was bonded to the grating layer. After an active modification process, the channel layer with top chamber was aligned and bonded to the grating layer with bottom chamber, and we obtained the final assemble optofluidic dye laser device.

Specifically, the detailed fabrication process is presented in Figure S1. The chamber molding master was prepared using negative photoresist SU-8 2075 via contact lithography with an EVG mask aligner (EVG 610, EV Group, St. Florian am Inn, Austria). Both the upper and bottom chambers, with the same height of 150 µm, were obtained by coating PDMS prepolymer (RTV 615, GE, 10:1 *w*/*w* ratio) onto the mold. For the creation of the 1.2 µm height flow channel, we employed an AZ 5214 photolithography approach. The grating mold (417 nm period, 50% pitch) was manufactured through nanoimprint lithography. Subsequently, the grating and flow layers, 30 µm in thickness, were achieved via spin-coating the PDMS prepolymer (RTV 615, GE, 5:1 *w*/*w* ratio) on their respective molds. To delicately peel off the thin film from the mold, we employed the double-layer PDMS method, followed by a longer thermal bonding process. After oxygen plasma treatment using a reactive ion etcher (RIE-10NR, Samco, Kyoto, Japan), the channel layer, coupled with the top chamber, was meticulously aligned; this was bonded to the grating layer housing the bottom chamber under a stereoscope (SZ61, Olympus, Tokyo, Japan). The entire bonded optofluidic dye laser device was obtained following a curing process at 80 °C overnight in an oven. Mirror treatment on the chip side walls was implemented for efficient dye excitation, following the approach outlined in Ref. [42].

### 2.3. Device Operation

Following the fabrication of the optofluidic device, Tygon tubing (Cole-Parmer, Vernon Hills, IL, USA) with stainless steel tubing (23G, New England Small Tube, Litchfield, CT, USA) was inserted into the device to inject the dye solution and regulate the vacuum in the side chambers. The gain medium, consisting of Rhodamine 6G (Rh6G, Exciton, Dayton, OH, USA) in a solution of dimethylsulfoxide (DMSO) and water, was injected into the microchannel in the flow layer.

To mechanically deform the grating layer, suction was applied through tubing connected to the vacuum chambers using an electronic vacuum regulator (ITV2090-312L5, SMC, Tokyo, Japan). The voltage input to the regulator was precisely controlled by a DC power supply (Agilent E3631A, Agilent Technologies, Santa Clara, CA, USA). The vacuum source connected to both the upper and bottom layers of the laser device generated a uniform stretching force on the grating membrane. Due to the exceptional stretchability of PDMS, the PDMS membrane with grating nanostructures could undergo approximately 50% deformation. The applied vacuum force on the hollow chambers adjacent to the grating thin layer caused stretching of the membrane, thereby increasing the grating period.

## 3. Results and Discussion

### 3.1. Laser Characterization

For the characterization of lasing, the optofluidic dye laser device was pumped using a 532 nm Nd:YAG laser of 5 ns duration and 1 Hz repetition rate (Elforlight, SPOT 10-200-532, Elforlight Limited, Daventry, UK). A longitude pumping scheme was selected to improve the pumping efficiency. The pump laser beam was focused on the side wall of the device by a 4× objective lens, forming a divergence beam near the focus point. To optimize light coupling into the grating structure, mirror treatment was applied to form optically flat side surfaces of the PDMS device. The laser output, collected by a 10× objective lens, was filtered at 532 nm using a notch filter and directed to an optical fiber connected to a spectrometer (Ocean Optics, HR2000+, Largo, FL, USA), as shown in Figure S2.

**Figure 3:**
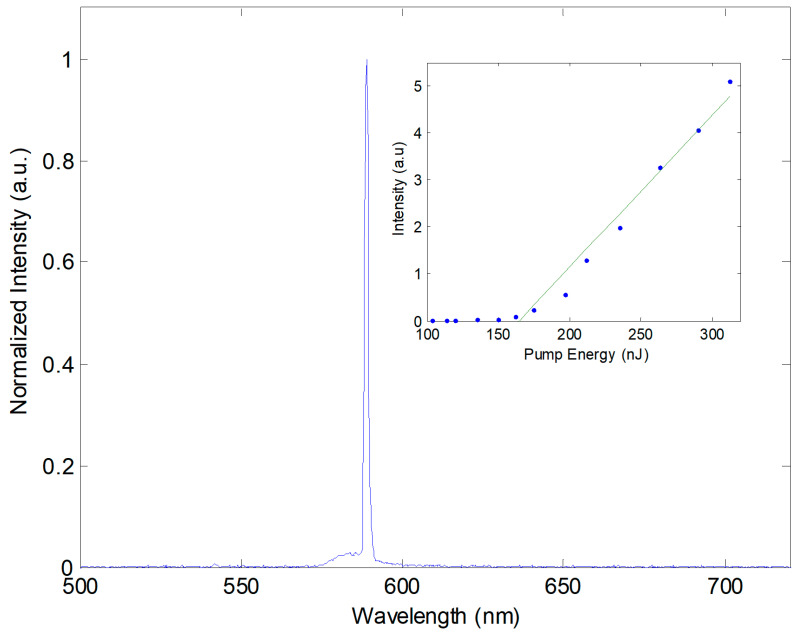
A typical emission spectrum of an optofluidic DFB dye laser. The inset shows the plots of laser output vs. pumping energy. The green line represents the fitting to the blue data points, used to determine the threshold energy.

First, we characterized the optofluidic dye laser device before tuning. A typical lasing spectrum above the pumping threshold is shown in Figure 3. The laser emission appears at the wavelength of 588.84 nm with a measured linewidth of 0.15 nm (limited by the spectrometer resolution). The inset depicts the plot of pumping pulse energy versus output pulse, revealing a threshold pump energy of 164.86 nJ/pulse.

The corrugated PDMS film forms a second-order slab waveguide DFB laser. Under the second-order Bragg condition, the resonance wavelength of the fabricated grating was determined by $\lambda =n_{eff}\Lambda$, where $n_{eff}$ is the effective index of the waveguide optical mode. With a grating period Λ = 417 nm, a high refractive index (RI) liquid containing RH6G dye is surrounded by low RI PDMS, forming a waveguide. Emission fluorescence in the resonance chamber containing the grating ensures the selection of the resonance wavelength, while other wavelengths are lost.

To achieve lasers with high output energies, a large gain volume is preferable. Thus, the flow channel containing the DFB grating was designed to be 3 mm in length and 20 μm in width. Lasing is induced by the longitude pumping of the gain medium in the DFB grating area. To optimize pumping efficiency, we carefully adjusted the angle between the waveguide and the pump beam axis. The dye solution concentration was adjusted, and a 2 mM Rh6G in a DMSO and water mixture (43% *v*/*v*) was used as the gain medium. Higher concentrations of the dye solution may decrease the quantum yield due to fluorescence quenching. To maintain single-mode output, the height of the chamber was controlled according to Equation (1):

(1) $$d_{c}=\lambda /\left(2\sqrt{n_{c}^{2}-n_{s}^{2}}\right)$$

where $d_{c}$ is the cutoff height of the chamber, $n_{c}$ is the RI of the core, $n_{s}$ is the RI of the surrounding materials, and λ is the resonance wavelength. To maintain a single-mode output in the resonance chamber, it is crucial to carefully control its height, as an increased height may result in the generation of multimode lasers. The difference between the RI of the core and the surrounding medium also plays a critical role in determining the number of modes produced. To achieve a single-mode laser output with a sufficiently high fluorescence intensity to provide feedback, we determined an optimal height of 1.2 μm, which satisfies the cutoff height, and a difference in RI of 0.008 that matches the $n_{eff}$ for the second-order Bragg condition.

A horizontal pump alone for the channel configuration was found to be more effective. According to the equation in Ref. [43], the threshold gain $g_{th}$ can be expressed as $g_{th}=a+a_{m}$, where a represents the propagation loss coefficient within the cavity, including factors such as optical scattering, absorption, and out-of-plane diffraction, and $a_{m}$ denotes the loss coefficient due to grating reflection. Compared to a vertical pump with only one excited spot, a horizontal pump excites more cavity volume, reducing the propagation loss coefficient a and the threshold gain $g_{th}$. The pump threshold power $P_{th}$ was determined by the threshold gain $g_{th}$, as illustrated in Equation (2):

(2) $$P_{th}=g_{th}Vh\nu /2\kappa \eta_{a}\;$$

where κ is the product of the confinement factor, singlet exciton lifetime, photoluminescence quantum yield, and stimulated emission cross-section, $\eta_{a}$ is the absorption efficiency of the pump light, *V* is the cavity volume, and hν  is the pump photon energy. Compared to a threshold energy of 78 nJ reported in Ref. [43], our configuration has three chambers, with two chambers used to stretch the grating film in the center chamber via vacuum. Smoothing treatment was applied to the first side wall to decrease the scattering loss, while two side walls in the first chamber could increase scattering loss. Consequently, the excited light could not be delivered completely to the resonance chamber, resulting in a higher threshold energy of 164.86 nJ.

In the second-order DFB laser, the laser can output in both the vertical and horizontal directions, making it suitable for diverse applications. Additionally, the *π*/2 phase shift can be omitted, simplifying the fabrication process and providing a convenient method for laser excitation in various applications.

### 3.2. Simulation Using FDTD Method

**Figure 4:**
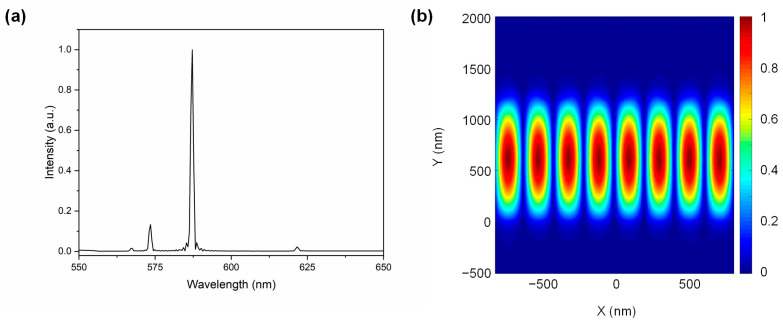
(**a**) Simulated spectrum of the DFB structure using the FDTD method. (**b**) Normalized magnetic field intensity at the resonance wavelength of 587.23 nm.

To understand the second-order DFB modes, an optical simulation was carried out using Lumerical software (8.19.1584), based on the finite-difference time-domain (FDTD) method. In these simulations, the laser produced by the fluorescent dye was modeled by placing multiple random electric dipole sources in the 550–650 nm range within the resonance chamber to excite the periodic unit cell structure. The refractive index of the fluorescence liquid in the resonance chamber was 1.420, and the refractive index of PDMS at 588 nm was 1.412, according to commercial datasheets. Two modes at the wavelengths of 573.50 nm and 587.23 nm were observed in the simulated spectrum in Figure 4a. A robust and narrow peak at 588 nm, consistent with experimental results, was observed, while the other resonance mode with a higher threshold energy was not observed in the experiment. The theoretical simulation suggested that the field distribution of the Eigen mode is predominantly confined within the resonance chamber with the active waveguide layer, as depicted in Figure 4b.

### 3.3. Laser Wavelength Tuning

For precise laser wavelength tuning, the grating membrane was stretched using four vacuum chambers integrated with the optofluidic device. In this setup, the top and bottom chambers were interconnected, and the air was evacuated from the left and right sides using two plastic tubes and two stainless steel needles, as shown in Figure S2. Vacuum force was applied to the four hollow chambers adjacent to the grating thin layer simultaneously, resulting in the stretching of the membrane and an increase in the grating period. We establish the correlation between pressure and membrane deformation through microscopic imaging, similar to the methodology depicted in Figure S3. This process involves correlating distinct vacuum levels (Δp) with the resulting alterations in the stretched membrane’s length (ΔL). This relationship is mathematically denoted as ΔL/L=k×Δp, as illustrated in Figure S4.

**Figure 5:**
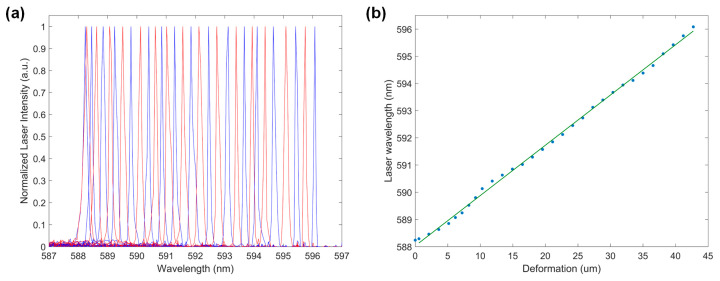
(**a**) Normalized lasing spectrum of the optofluidic DFB dye laser at 32 different wavelengths within a tuning range of 7.84 nm. (**b**) The relationship of the output laser wavelength vs. membrane deformation, illustrated by the blue dots, with the fitting indicated by the green line.

A normalized lasing spectrum at 32 different wavelengths was demonstrated in Figure 5a, showcasing highly robust and flexible tunability. The laser emission spectra graph demonstrated a tuning range of 7.84 nm, covering wavelengths from 588 nm to 596 nm in a single-mode operation. Although the PDMS membrane is capable of producing up to ~50% deformation, only 1.28% deformation was utilized to ensure single-mode lasing operation. If the stretched grating period falls outside the range, the supported resonance does not overlap with the gain medium, and the lasing appears to be very weak.

Highly accurate laser wavelength tuning was achieved through precisely controlled vacuum force-induced membrane deformation. The electronic tuning of the vacuum regulator enabled a small increment of 0.8 kPa at each tuning step, corresponding to an average laser wavelength tuning of 0.25 nm. Therefore, a tunable laser device was obtained using a pneumatic tuning mechanism with a high tuning resolution, flexible operation, and fast tuning response.

Figure 5b demonstrates the relationship between the lasing wavelength and membrane deformation, while the membrane deformation increased linearly as a function of the applied vacuum force. Experimental data indicated an average increment rate of ~1.8 μm/kPa for the total grating length during membrane stretching. We observed that, as the applied vacuum increased, the output laser wavelength increased linearly as a function of the membrane deformation. This integrated laser in a microfluidic chip provides an alternative to tunable lasers for constructing compact coherent sources. Additionally, the second-order mode produces both vertical and horizontal lasers, enhancing versatility for microfluidic chip applications.

By adjusting the vacuum force applied to the laser device, the tunable optofluidic dye laser could be easily tuned back and forth. This resulted in a robust integrated optofluidic laser device with highly accurate and flexible wavelength tuning. The on-chip circulation of dye can be achieved by pumping dye solution in and out of the flow channel. Constructing a highly accurate tunable laser with DFB configuration presents the opportunity to develop a fully integrated optofluidic system for biosensing and spectroscopy applications.

## 4. Conclusions

In conclusion, we have demonstrated a highly accurate pneumatically tunable optofluidic dye laser with a distributed feedback configuration. Flexible laser wavelength tuning was achieved at a high resolution of 0.25 nm in a 7.84 nm tuning range in single-mode operation. The optofluidic laser device exhibited strong emission and low-threshold lasing (164 nJ/pulse). The tunable laser in the visible spectral range exhibits favourable properties and can be widely used in many biosensing applications that require highly sensitive tuning operation. We introduced a multilayer soft lithography process that enables the fabrication of tunable optofluidic laser devices. The tunable optofluidic dye lasers, with their low manufacturing cost and a wide range of emission wavelengths, have the potential to be used as a novel optical toolbox for lab-on-a-chip applications such as biosensing and spectroscopy.

## Data Availability

The data that support the findings of this study are available from the corresponding author upon reasonable request.

## Supplementary Materials

The following supporting information can be downloaded at: https://www.mdpi.com/article/10.3390/mi15010068/s1, Figure S1. The detailed fabrication process for the optofluidic dye laser device. Figure S2. The experiment setup of the tunable optofluidic dye laser, including the pumping laser, optofluidic dye laser device, the vacuum regulator, and the fiber spectrometer. Insert: The photograph of the optofluidic dye laser device. Figure S3. The variation of the stretched membrane under vacuum pressure. Scale bar: 50 µm. Figure S4. The membrane deformation is a function of the vacuum pressure. Table S1. The geometrical dimensions of the device. Table S2. The refractive indices of the materials.
