# Supplementary material for: Highly Accurate Pneumatically Tunable Optofluidic Distributed Feedback Dye Lasers
Source: Micromachines (Basel). 2023 Dec 29;15(1):68. doi: 10.3390/mi15010068 (PMC10820426; doi:10.3390/mi15010068)
Supplement: Supplementary file 1 [file micromachines-15-00068-s001.zip › micromachines-2767799-supplementary.pdf]

micromachines

Supplementary Information

Highly Accurate Pneumatically Tunable Optofluidic Distributed Feedback Dye Lasers

Hongtao Feng, Jiaxin Zhang, Weiliang Shu, Xiaosong Bai, Liang Song and Yan Chen *

Shenzhen Institute of Advanced Technology, Chinese Academy of Sciences, Shenzhen 518055, China; ht.feng@siat.ac.cn (H.F.); jx.zhang1@siat.ac.cn (J.Z.); wl.shu@siat.ac.cn (W.S.); xs.bai@siat.ac.cn (X.B.); liang.song@siat.ac.cn (L.S.)
* Correspondence: yan.chen@siat.ac.cn; Tel.: +86-(755)-8639-2254

S1: The Detailed Fabrication Process for the Optofluidic Dye Laser Device

We fabricated the chamber master mold via contact photolithography with an EVG mask aligner using SU-8 2075 photoresist. Both the upper and bottom PDMS chambers (150 μm in height) were molded from this master. The flow channel (1.2 μm in height) was defined by photolithography using AZ 5214 photoresist. The grating mold, with a period of 417 nm and a duty cycle of 50%, was produced through nanoimprint lithography. To create the grating layer and the flow channel layer (30 μm thick), PDMS prepolymer was spin-coated and then delicately peeled off their molds using a double-layer technique. Following oxygen plasma activation, Stereoscopic alignment was used to align and bond the flow layer to the grating layer, with the chambers aligned accordingly. Finally, we completed the device fabrication by thermally bonding the components at 80°C overnight.

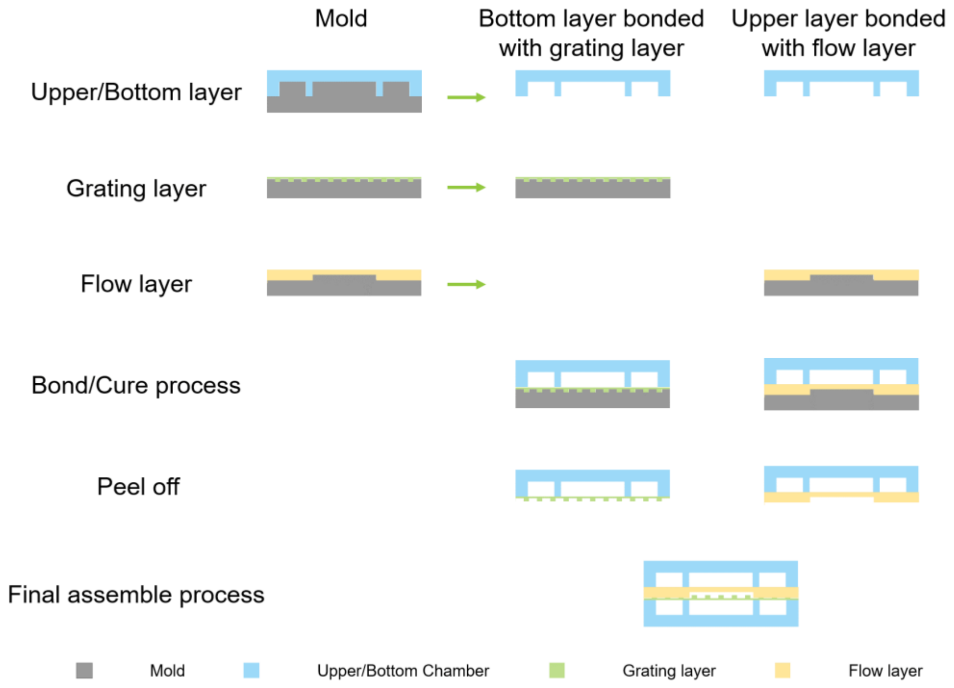

Figure S1. The detailed fabrication process for the optofluidic dye laser device.

S2: The Experiment Setup of Tunable Optofluidic Dye Laser

The pump laser was focused into the channel through a 4× objective lens. Concurrently, the emission from the dye laser, collected by a 10× objective, was passed through a 532 nm notch filter to block the pump light and enable observation of the generated laser emission signal, prior to coupling into an optical fiber connected to a spectrometer for analysis. Vacuum force applied to the two side chambers adjacent to the grating thin layer simultaneously via stainless steel tubing, resulting in stretching of the membrane and an increase in the grating period. Through this approach, the desired tunable optofluidic dye laser output was obtained.

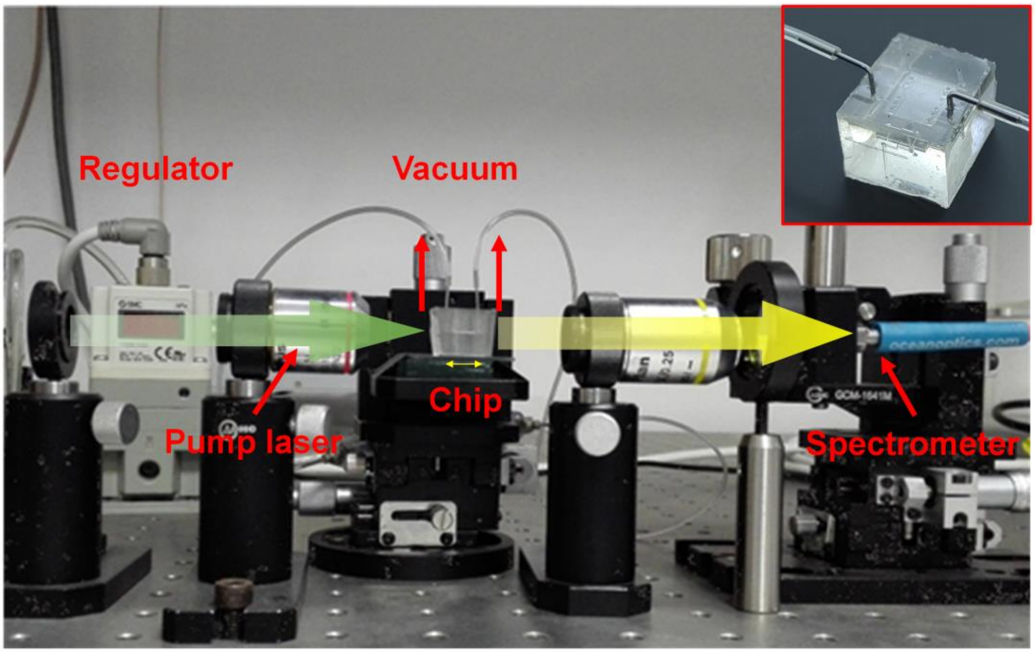

Figure S2. The experiment setup of the tunable optofluidic dye laser, including the pumping laser, optofluidic dye laser device, the vacuum regulator, and the fiber spectrometer. Insert: The photograph of the optofluidic dye laser device.

S3: Stretchable Membrane under Vacuum Pressure

Membrane deformation was monitored using microscopic imaging under different vacuum pressure. Distinct changes in the membrane morphology before and after the stretching process are observable.

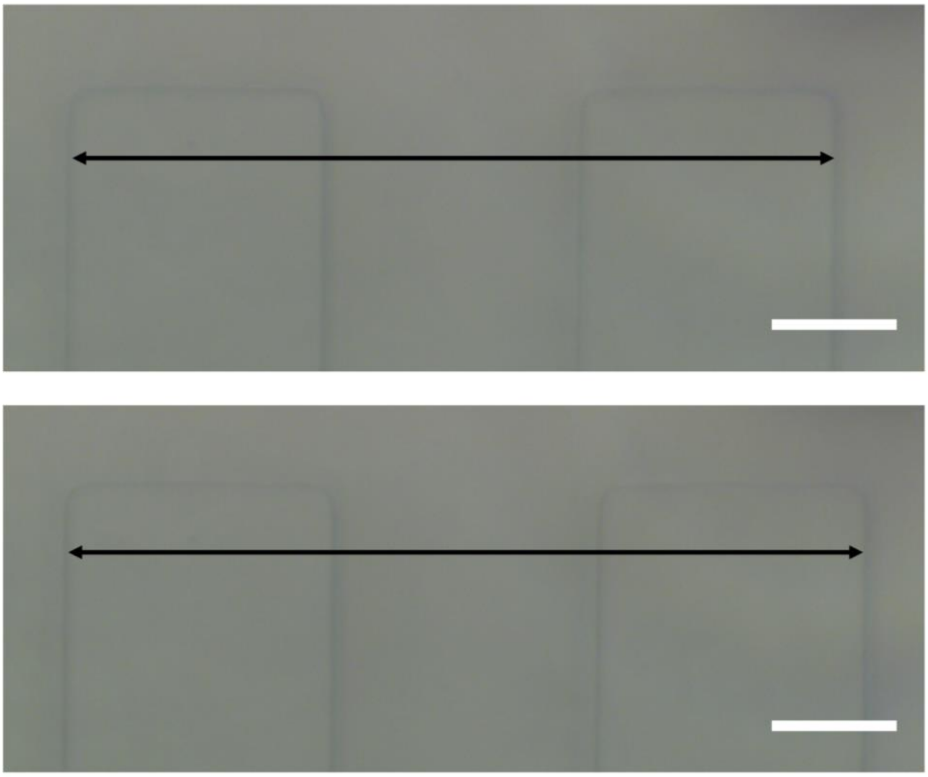

Figure S3. The variation of the stretched membrane under vacuum pressure. Scale bar: 50 μm.

S4: Deformation under Different Vacuum Pressure

The relationship between applied pressure and membrane deformation was established. This process involves correlating distinct vacuum levels (Δp) with the resulting alterations in the stretched membrane's length (ΔL). This relationship is mathematically denoted as $\Delta L/L = k \times \Delta p$.

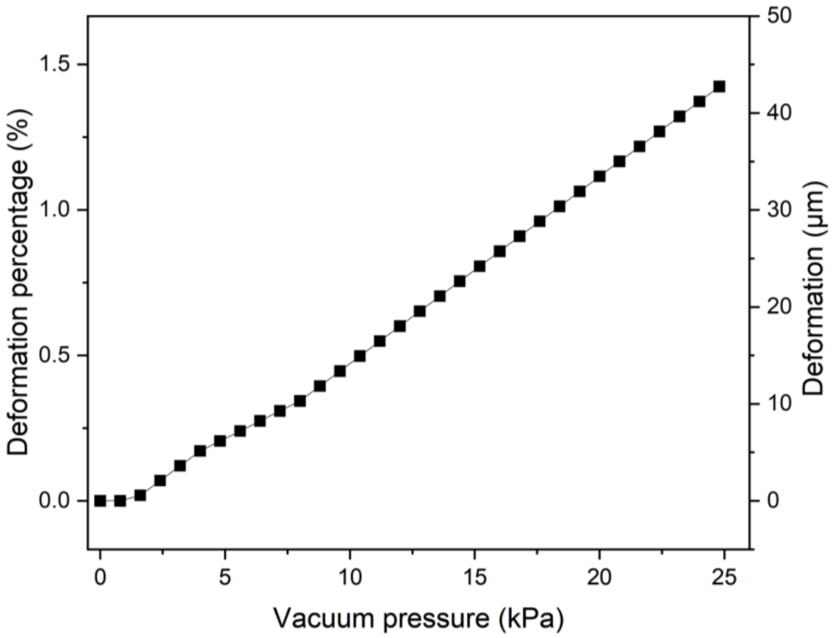

Figure S4. The membrane deformation is a function of the vacuum pressure.

S5: The Geometrical Dimensions of the Device and the Refractive Indices of the Materials

Table S1. The geometrical dimensions of the device.

Name	Parameters
Height of chamber	150 μm
Side wall thickness of chamber	75 μm
Thickness of flow layer	30 μm
Height of flow channel	1.2 μm
Length of the flow channels	3 mm
Thickness of grating layer	30 μm
Grating dimensions	100 nm depth, 417 nm period, 50% pitch

Table S2. The refractive indices of the materials.

Name	Refractive Indices
Fluorescence liquid	1.420
PDMS	1.412
